# Development of a Mathematical Model of the Self-Shielded Flux-Cored Arc Surfacing Process for the Determination of Deposition Rate

**DOI:** 10.3390/ma17225616

**Published:** 2024-11-17

**Authors:** Michał Szymura, Artur Czupryński, Vladislav Ochodek

**Affiliations:** 1 FPM S.A., Wyzwolenia Street 12C, 43-190 Mikołów, Poland; mich.szymura@gmail.com; 2 Department of Welding Engineering, Faculty of Mechanical Engineering, Silesian University of Technology, Konarskiego 18A, 44-100 Gliwice, Poland; 3 Department of Mechanical Technology, Faculty of Mechanical Engineering, VSB–Ostrava Technical University of Ostrava, 17. listopadu 2172/15, 708 00 Ostrava-Poruba, Czech Republic; vladislav.ochodek@vsb.cz

**Keywords:** surfacing, mathematical model, deposition rate, three-level design, flux-cored wire

## Abstract

The article presents a method of developing a mathematical model of the arc surfacing process performed using the self-shielded flux-cored filler metal wire with the chromium cast iron (Fe15) weld deposit. A three-level design (static, determined, and complete) was used to determine the function of the test object, thus enabling the simulation of deposition rate in relation to wire feed speed and electrode extension. The deposition rate for the specified set of surfacing parameters amounted to between 4.31 kg/h and 11.25 kg/h. The study was also concerned with identifying the effect of the significance level of test factors and interactions between them on the resultant factor, as well as an assessment of the adequacy of the test object function. In relation to significance level α = 0.01, regression coefficients b_0_, b_1_, b_2_, and b_11_ significantly affected the deposition rate of the surfacing process. Coefficient b_22_ was significant at a level of 0.40, whereas coefficient b_12_ was significant at a level of 0.15. The mathematical model presenting the effect of wire feed speed and electrode extension, as well as interactions between them on the deposition rate of the surfacing process, was adequate for the adopted level of significance α = 0.05.

## 1. Introduction

Surfacing processes are used to produce new elements (preventive surfacing) [1,2,3,4], refurbish worn elements (repair surfacing) [5,6,7,8], and rapid prototyping [9,10,11]. The industrial application of surfacing technologies depends primarily on quality-related and economic criteria [4,12,13,14,15,16,17,18,19,20]. In general, when determining the total cost of surfacing processes (as well as welding), such components as labor cost, material cost, equipment cost, and electrical power cost should be considered. Labor and overhead costs can constitute up to 85% of the total costs of a given welding process. The cost of welding consumables, on the other hand, is about 8–15% of the total cost. In the analysis concerning costs of surfacing with the self-shielded flux-cored filler metal wire, deposition rate constitutes a variable when calculating the cost of labor, the filler metal wire (in the group of material), investments, depreciation, and repairs (in the group of equipment-related costs) [17,21].

**Figure 1:**
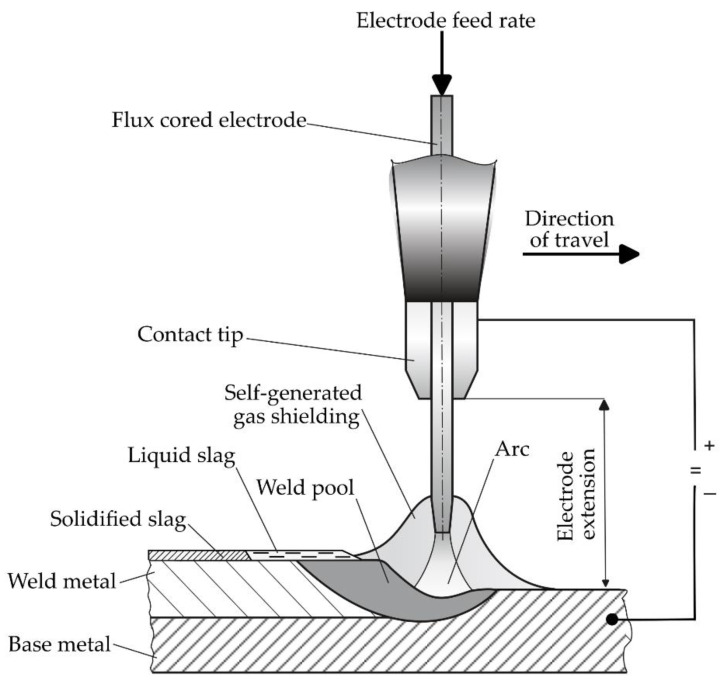
Self-shielded flux cored arc surfacing.

The deposition rate of flux-cored arc surfacing depends primarily on the properties of the flux-cored filler metal wire (e.g., components of the flux mixture, the construction of the flux-cored wire, etc.) and surfacing technology [22,23,24,25,26,27,28,29,30]. The deposition rate of automated self-shielded flux-cored arc surfacing is influenced by, among other things, the value of current and electrode extension [23,24,25,30]. The value of current changes along with changes in the filler metal wire feed speed while maintaining the constant values of the remaining surfacing process parameters. In relation to a given wire feed speed, a change in electrode extension leads to a change in the value of current [31]. The process of self-shielded flux-cored arc surfacing is shown in Figure 1.

Research [25] concerning the self-shielded flux-cored arc surfacing performed using flux-cored filler metal wires from alloy groups Fe1, Fe9, ZFe13, and Fe15 revealed that an increase in current was accompanied by an increase in deposition rate, filler metal wire losses (due to spatter), burning, and slag formation. The Authors of the publication did not provide information concerning a method enabling the determination of both the above-named surfacing process indicators.

Publication [23], which concerns the effect of selected parameters of surfacing with Fe7 flux-cored filler metal wire, stated that, in terms of the conventional arc (i.e., non-pulsed), an increase in current was accompanied by an increase in deposition rate and weld deposit efficiency. In cases of changes in electrode extension, the highest deposition rate was obtained in relation to the central level, whereas the lowest efficiency was achieved in relation to the upper level. An increase in electrode extension led to an increase in weld deposit efficiency. The publication did contain information related to a method enabling the determination of deposition rate.

Tests [30] concerning the effect of, among others, current and electrode extension in the surfacing process performed using the ZFe13 self-shielded flux-cored filler metal wire on deposition rate revealed that an increase in the above-named parameters increased deposition rate. The level of effect significance was higher in relation to the current. An increase in current and electrode extension reduced percentage losses due to spatter, burning, and slag formation. The deposition rate was calculated as the product of the mass of the weld deposit (in accordance with the ISO 2401 [32] standard) and its deposition time.

The work [24] concerning the analysis of selected parameters of the surfacing process performed using the Fe15 self-shielded flux-cored filler metal wire revealed, regardless of the type of current and its polarity, an increase in current increased deposition rate. When using direct current with reversed polarity, an increase in current was accompanied by increased weld deposit efficiency. In relation to straight polarity, changes in current did not significantly affect weld deposit efficiency. An increase in electrode extension led to a decrease in weld deposit efficiency, and the aforesaid effect was statistically relevant.

Available reference publications present research results concerning the effect of current and electrode extension on the deposition rate of the self-shielded flux-cored arc surfacing process [23,24,25,30]. The influence of the above-named parameters was considered, assuming the lack of interactions between them. However, reference publications did not contain quantitative data specifying the significance of the effect of interaction between wire feed speed and electrode extension on deposition rate. Similarly, there was no data available that made it possible to compare the effect of the aforesaid process parameters on the course of the surfacing process and the relationship between them. In other words, as of today, there is no mathematical model describing the above-named correlations.

In view of the foregoing, the Authors of this research work undertook to develop a mathematical model of the self-shielded flux-cored arc surfacing process (involving the use of the HARDFACE HC-O filler metal wire), making it possible to identify the significance of the effect of wire feed speed and electrode extension, as well as the correlation between them, on the deposition rate. In addition, the Authors assessed the adequacy of the test object function. The knowledge of the surfacing process-related mathematical model could be used to simulate the effects of the aforesaid process.

## 2. Test Materials

Arc surfacing tests were performed using the HARDFACE HC-O flux-cored filler metal wire (Welding Alloys) with a diameter of 2.8 mm and ensuring the obtainment of the weld deposit of high-alloy chromium cast iron (type Fe-Cr-C, group Fe15, in accordance with EN 14700 [33]). The chemical composition of the filler metal wire and post-surface hardness provided by the manufacturer are presented in Table 1 [34]. The HARDFACE HC-O filler metal wire is recommended, among other things, for the protection of surfaces exposed to intense metal-mineral abrasive wear combined with moderate impact loads. The base material was in the form of plates with dimensions of 300 mm × 75 mm × 12 mm (in accordance with ISO 2401 [32]), cut out of unalloyed steel S355JR, in accordance with the EN 10025-2 [35] standard.

## 3. Tests

### 3.1. Mathematical Modelling of the Self-Shielded Flux-Cored Arc Surfacing Process

The objective of the determination of the mathematical model of the self-shielded flux-cored arc surfacing process was to identify the effect of the filler metal wire feed speed and electrode extension, as well as correlations between the factors on the deposition rate. The development of the mathematical model of the surfacing process required the performance of experimental tests involving 7 stages, i.e.,:

− identification of sets of test, resultant, constant, and confounding (disturbing) factors;
− determination of the variability range of factors subjected to tests;
− adoption of the class of the mathematical model of the test object;
− planning and performing the experiment;
− identification of the test object function;
− identification of the significance of the effect of test factors and their correlations on the resultant factor;
− assessment of the adequacy of the test object function.

### 3.2. Identification of Sets of Test, Resultant, Constant, and Confounding Factors

The deposition rate of automated arc surfacing was performed using a given type of self-shielded flux-cored filler metal wire depending on the diameter of the filler metal wire, current type, and polarity, as well as electrode extension [23,24,25,30,31].

**Figure 2:**
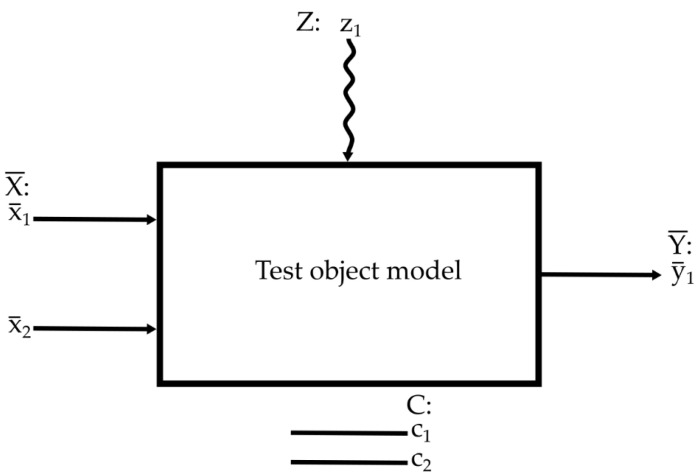
Test object model ($\bar{X}$—set of factors subjected to tests, x_1_—electrode feed rate, x_2_—electrode extension, y_1_—deposition rate (resultant factor), C—set of constant factors: c_1_—filler metal wire diameter, c_2_—current type and polarity, and z_1_—random fluctuations of surfacing parameters (confounding factor)).

Within the scope of the research work, factors subjected to testing included wire feed speed and electrode extension, where deposition rate was a resultant factor. Constant factors included the diameter of the filler metal wire, as well as the type of current and its polarity. Random fluctuations of surfacing parameters were confounding factors. The schematic diagram of the test object model, including the classification of factors, is presented in Figure 2.

### 3.3. Determination of the Variability Range of Factors Subjected to Tests

The range of the variability of wire feed speed and that of electrode extension was determined based on the technological recommendations formulated by the manufacturer of the filler metal wire [34] and the results of initial surfacing tests. The criterion of acceptability (of a given set of parameters) was the stability of the surfacing process, ensuring the obtainment of imperfection-free overlay welds. For this reason, the area of tests was determined as follows:

− electrode feed rate (x_1_) restricted within the range of 3.00 m/min to 6.50 m/min;
− electrode extension (x_2_) restricted within the range of 25 mm to 35 mm.

### 3.4. Adoption of the Class of the Mathematical Model of the Test Object

In accordance with the objective of the research work, the mathematical model of the test object should take into account interactions between factors subjected to the tests. In addition, because of the possible occurrence of non-linear correlations, the selection was restricted to models determined using programs higher than two-level ones [30].

Taking the foregoing into account, the Authors planned the performance of the experiment using a three-level design (static, determined, and complete), enabling the determination of the test object function in the form of the second-degree polynomial with interactions between the factors subjected to the tests [36]:

(1) $$\hat{\bar{y}}=b_{0}+b_{1}{\bar{x}}_{1}+b_{2}{\bar{x}}_{2}+b_{11}{\bar{x}}_{1}^{2}+b_{22}{\bar{x}}_{2}^{2}+b_{12}{\bar{x}}_{1}{\bar{x}}_{2}$$

where:

$\hat{\bar{y}}$—resultant factor (measured parameter);
b_0_; b_1_; b_2_; b_11_; b_22_; b_12_—regression coefficients.

### 3.5. Plan of the Experiment

In accordance with the plan of three-level design performance, the values of the factors subjected to the tests were determined on three levels. The upper and lower levels of the test factors were identified based on the recommendations formulated by the manufacturer of the filler metal wire [34] and results obtained in surfacing tests. The central level was the mean of the above-named values. The adopted values of factors tested on the three above-named levels are presented in Table 2.

In accordance with the assumptions of the three-level program, encoded variables amounted to the following:

(2) $${\overset{ˇ}{\bar{x}}}_{1}=\frac{x_{1}-\left(\frac{x_{1max}+x_{2max}}{2}\right)}{\frac{x_{1max}-x_{2max}}{2}}=\left(\frac{x_{1}-4.75}{1.75}\right)$$

(3) $${\overset{ˇ}{\bar{x}}}_{2}=\frac{x_{2}-\left(\frac{x_{2max}+x_{2max}}{2}\right)}{\frac{x_{2max}-x_{2max}}{2}}=\left(\frac{x_{2}-30}{5}\right)$$

The plan of the experiment is presented in Table 3. Three parallel measurements were performed for each system of values of factors subjected to tests. It was assumed that the value of the resultant factor should correspond to the arithmetic mean of parallel measurements. The regression coefficients of the model were calculated using Formula (4).

(4) $$b=(X^{T}X{)}^{-1}X^{T}Y$$

where:

b—given regression coefficient;
X—matrix of input variables;
(X^T^X)^−1^—matrix of covariances;
Y—vector of mean experiment results.

The surfacing tests were performed using a Multi Surfacer D2 automated surfacing station (Welding Alloys) featuring a microprocessor control system, enabling the setting of required travel speeds and the repeatable positioning of the welding head. One hard-facing bead with a length of 180 mm was made for each specimen. The technological parameters of the surfacing process are presented in Table 4. Before and after the surfacing process, the specimens were weighed using a laboratory balance with an accuracy of up to 0.01 g. Weighing was preceded by the cleaning and removal of spatter from the specimens. The deposition rate of the surfacing process was determined based on the difference in the specimen mass and the duration of the surfacing process.

### 3.6. Experiment Results

The results of the experiment, performed in accordance with the three-level program, are presented in Table 5.

### 3.7. Elimination of Gross Errors

It was assumed that the distribution of the identified values of deposition rate was normal. In order to reject measurement results encumbered with gross errors, hypothesis H was verified against alternative hypothesis K. The adopted level of significance amounted to 0.05. Because of the lack of knowledge concerning expected value μ_i_ and variance σ^2^, the K^+^ and K^−^ hypotheses were verified using statistics $B_{6}^{+}$ and $B_{6}^{-}$. Statistics $B_{6}^{+}$ and $B_{6}^{-}$ (Table 6) were calculated using Formulas (5) and (6):

(5) $$B_{6}^{+}=\frac{y_{3}-y_{2}}{y_{3}-y_{1}}$$

(6) $$B_{6}^{-}=\frac{y_{1}-y_{2}}{y_{3}-y_{1}}$$

where:

y_1_; y_2_; y_3_—experiment results (y) arranged in a non-decreasing sequence, y_1_ ≤ y_2_ ≤ y_3_.

The calculated absolute values of statistics $B_{6}^{+}$ and $B_{6}^{-}$ were lower than the critical value of statistics $B_{6(0.05;3)}^{+}$; hence, there are no grounds for the rejection of both maximum and minimum values obtained in the measurements performed.

### 3.8. Calculation of Coefficients as a Function of Regression and the Identification of the Test Object Function

The regression coefficients of the test object function were calculated in accordance with the schematic diagram of the three-level design performance and based on obtained measurement results. The determined function of the test object is expressed by Equation (7):

(7) $$\hat{y}=7.4804+3.0189{\hat{x}}_{1}+0.1967{\hat{x}}_{2}+0.2078{\hat{x}}_{1}^{2}+0.0344{\hat{x}}_{2}^{2}-0.0600{\hat{x}}_{1}{\hat{x}}_{2}$$

### 3.9. Verification of the Homogeneity of Variances in the Test

The homogeneity of variances in the test was verified using Cochran’s Q test, enabling the comparison of variances in relation to the identical number of parallel measurements for all combinations of test plan levels. The hypothesis of the equality of variances was verified at significance level α = 0. The calculated value of Cochran’s Q test (0.3876) was lower than critical value G_0.05;9;2_; therefore, there was no basis for the rejection of the hypothesis concerning the homogeneity of variances.

### 3.10. Assessment of the Significance of the Regression Coefficients of the Test Object Function

The verification of the significance of the effect of the test factors on the resultant factor necessitated the verification of the significance of the identified regression coefficients of the model. The assessment of significance was performed using Student’s *t*-test. In relation to each regression coefficient, it was necessary to determine the level of the significance of effect, for which a given coefficient was significant (Table 7). It was decided that the lowest verified level of significance was 0.01.

### 3.11. Assessment of the Adequacy of the Test Object Function

The adequacy of the test object function (determined on the basis of the three-level design) in relation to the actual object was verified using the F-test (Equation (8); Table 8). The adopted level of significance was 0.05. The variance of adequacy (Equation (9)) was calculated using the values of the initial factor obtained from the model and based on experiment results (Table 5).

(8) $$F=\frac{s_{ad}^{2}(y)}{s^{2}(y)}$$

where:

$s_{ad}^{2}(y)$—variance of adequacy.

(9) $$s_{ad}^{2}\left(y\right)=\frac{r\sum_{i=1}^{N}({{\hat{y}}_{i}-y_{i})}^{2}}{N-k-1}$$

where:

${\hat{y}}_{i}—$value of the *i*th resultant factor calculated on the basis of the mathematical model;
$y_{i}—$value of the *i*th resultant factor obtained in the experiment;
k—number of regression equation terms (including the free term).

The value of the F-test, calculated on the basis of the statistical analysis of test results (Table 8), was lower than critical value F_0.05;2;18_ of the F-test. The foregoing justified the formulation of the conclusion that, for the adopted level of significance and calculated numbers of degrees of freedom, the developed test object function was adequate within the specified range of changes in the values of test factors.

### 3.12. Development of the Mathematical Model

**Figure 3:**
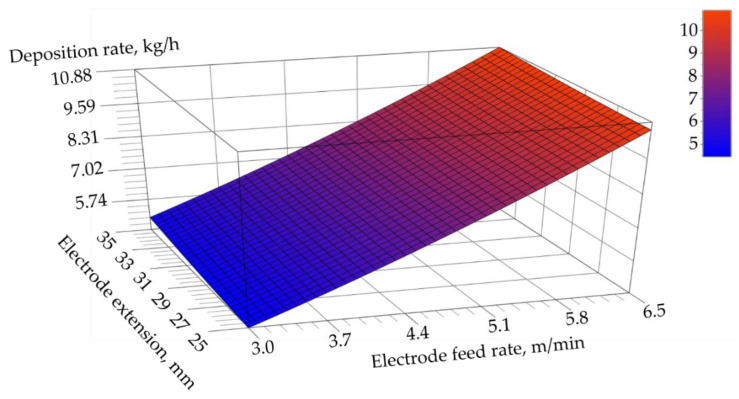
Graphic representation of the mathematical model describing the dependence of the electrode feed rate and electrode extension on the deposition rate of the self-shielded flux-cored surfacing process performed using the HARDFACE HC-O filler metal wire.

After decoding variables ${\hat{x}}_{1}$ and ${\hat{x}}_{2}$ and assessing the adequacy of the test object function, the mathematical model describing the deposition rate of the self-shielded flux-cored arc surfacing process could be expressed using Equation (10) and illustrated graphically (Figure 3):

(10) $$\hat{y}=7.4804+3.0189\times \left(\frac{x_{1}-4.75}{1.75}\right)+0.1967\times \left(\frac{x_{2}-30}{5}\right)+0.2078\times {\left(\frac{x_{1}-4.75}{1.75}\right)}^{2}+0.0344\times {\left(\frac{x_{2}-30}{5}\right)}^{2}-0.0600\times \left(\frac{x_{1}-4.75}{1.75}\right)\times \left(\frac{x_{2}-30}{5}\right)$$

## 4. Analysis of Test Results

The objective of the study was to identify the significance of the effect of wire feed speed and electrode extension, as well as their correlations on the deposition rate of the self-shielded flux-cored arc surfacing process.

The planning of the experiment using the three-level design (static, determined, and complete) enabled the development of a mathematical model describing the effect of the above-named parameters and the interactions between them. The concept of the mathematical model of the surfacing process (assumed before the tests) was confirmed experimentally; the model proved adequate within the range of changes in previously adopted surfacing parameters. The knowledge of the mathematical model of the surfacing process could help simulate the effects of process performance.

The assessment of the significance of regression coefficients of the test object function led to the conclusion that, in relation to significance level α = 0.01, coefficients b_0_, b_1_, b_2_, and b_11_, significantly affected the deposition rate of the surfacing process. Coefficient b_22_ was significant at a level of 0.40, whereas coefficient b_12_ was significant at a level of 0.15. In the Authors’ opinion (based on extra-statistical premises connected with the test object), regarding the regression coefficients of the free term (b_0_), both terms connected with the filler metal wire feed rate (b_1_, b_11_) and the first-power term concerning electrode extension (b_2_) was significant. In turn, the coefficients of the second power of the term concerning electrode extension (b_22_) and interaction between the test factors (b_12_) had nearly no significant effect on the deposition rate of the surfacing process.

The mean deposition rate for the specified set of surfacing parameters amounted to between 4.45 kg/h and 10.88 kg/h, whereas the range amounted to 0.71 kg/h. In relation to the given electrode extension, an increase in wire feed speed was accompanied by an increase in the mean deposition rate. Similarly, the deposition rate of the surfacing process was affected by electrode extension. Most probably, the extremum of the deposition rate was not reached within the specified range of changes in self-shielded flux-cored arc surfacing process parameters.

The differences in the mean deposition rate of the surfacing process between the identified electrode feed rate levels amounted to 6.05 kg/h (lower level of electrode extension), 6.26 kg/h (central level of electrode extension), and 5.81 kg/h (upper level of electrode extension). In relation to the differences between electrode extension levels, the above-named values amounted to 0.63 kg/h (lower level of electrode feed rate), 0.17 kg/h (central level of electrode feed rate), and 0.39 kg/h (upper level of electrode feed rate). The differences in the mean deposition rate for the adopted electrode extension were significantly lower than those related to wire feed speed. The foregoing was confirmed in the values of the regression coefficients of the mathematical model. Both coefficients concerning electrode extension (b_2_ and b_22_) were lower than those of wire feed speed (b_1_ and b_11_).

The force of dispersion expressed using the range related to given levels of test factors was restricted between kg/h 0.04 and 0.71 kg/h. The above-named dispersion could result from the changes in the characteristics of flux-cored wire melting in relation to a given set of surfacing process parameters (e.g., different filler metal wire losses due to spatter and, consequently, weld deposit efficiency), the measurement of the intermediate resultant factor (deposition rate expressed in kg/h), and random fluctuations of surfacing process parameters.

**Figure 4:**
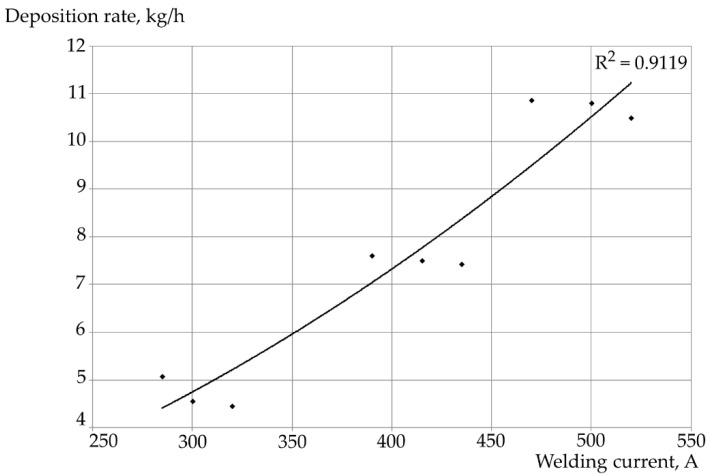
Correlation between the deposition rate of self-shielded flux-cored arc surfacing performed using the HARDFACE HC-O filler metal wire and current in relation to the line of regression being the second-degree polynomial.

An increase in electrode feed rate was accompanied by an increase in welding current. A decrease in electrode extension was accompanied by an increase in current [31]. The analysis of the effect of current on deposition rate revealed the relatively high conformity of the determined regression lines with the empirically obtained values (Figure 4). Determination coefficient R^2^ for the polynomial dependence (of the second degree) amounted to 0.9119. In turn, the coefficient of determination in relation to the linear dependence amounted to 0.9078.

## 5. Conclusions

The self-shielded flux-cored arc surfacing tests performed using the HARDFACE HC-O filler metal wire justified the formulation of the conclusions presented below.

1. The mathematical model presenting the effect of wire feed speed and electrode extension, as well as interactions between them on the deposition rate of the surfacing process, was adequate for the adopted level of significance (α = 0.05).
2. In relation to significance level α = 0.01, the coefficients of regression of the test object function b_0_ (of the free term), b_1_ (of the term concerning electrode feed rate in the first power), b_2_ (of the term concerning electrode extension in the first power), and b_11_ (of the term concerning electrode feed rate in the second power) had a significant effect on the deposition rate of the surfacing process. Coefficient b_22_ (of the term concerning electrode extension in the second power) was significant at a level of 0.40. In turn, coefficient b_12_ (of the term concerning the interaction between the wire feed speed and electrode extension) was significant at a level of 0.15.

## Figures and Tables

**Table 1:** Chemical composition of the weld deposit of the HARDFACE HC-O flux-cored filler metal wire and post-surface hardness [34].

Chemical Composition of the Weld Deposit, wt%					Hardness of the Third Overlay Weld Layer, HRC
C	Cr	Si	Mn	Fe	
5.0	27.0	1.5	1.5	Bal.	58–64

**Table 2:** Values of test factor levels.

Test Factor	$\mathrm{Electrode}\;\mathrm{Feed}\;\mathrm{Rate}\;({\bar{x}}_{1}$), m/min	$\mathrm{Electrode}\;\mathrm{Extension}\;({\bar{x}}_{2}$), mm
Upper level	6.50	35
Central level	4.75	30
Lower level	3.00	25

**Table 3:** Plan of the experiment.

Experiment No. (n)	$\mathrm{Encoded}\;\mathrm{Variable}\;({\overset{ˇ}{\bar{x}}}_{1}$)	$\mathrm{Encoded}\;\mathrm{Variable}\;({\overset{ˇ}{\bar{x}}}_{2})$	Experiment Result (y)
1	−	−	y_1_
2	0	−	y_2_
3	+	−	y_3_
4	−	0	y_4_
5	0	0	y_5_
6	+	0	y_6_
7	−	+	y_7_
8	0	+	y_8_
9	+	+	y_9_

**Table 4:** Parameters of self-shielded flux-cored arc surfacing performed with the HARDFACE HC-O filler metal wire.

Experiment No.	Electrode Feed Rate, m/min	Electrode Extension, mm	Current, A
1	3.00	25	320
2	4.75	25	435
3	6.50	25	520
4	3.00	30	300
5	4.75	30	415
6	6.50	30	500
7	3.00	35	285
8	4.75	35	390
9	6.50	35	470

Notes: the surfacing was performed in the flat position (PA) using direct current with straight polarity, an arc voltage of 30.0 V, and a travel speed of 0.54 m/min.

**Table 5:** Test results concerning the deposition rate of the surfacing process performed using the HARDFACE HC-O filler metal wire.

Experiment No. (n)	$\mathrm{Encoded}\;\mathrm{Variable}\;({\overset{ˇ}{\bar{x}}}_{1}$)	$\mathrm{Encoded}\;\mathrm{Variable}\;({\overset{ˇ}{\bar{x}}}_{2})$	Experiment Result (y)			
			1	2	3	Mean
1	−	−	4.62	4.42	4.31	4.45
2	0	−	7.37	7.49	7.41	7.42
3	+	−	10.29	10.64	10.56	10.50
4	−	0	4.72	4.51	4.42	4.55
5	0	0	7.43	7.35	7.71	7.50
6	+	0	10.64	11.25	10.54	10.81
7	−	+	4.90	4.96	5.37	5.08
8	0	+	7.61	7.57	7.59	7.59
9	+	+	10.74	11.13	10.78	10.88

**Table 6:** Calculated values of statistics $B_{6}^{+}$ and $B_{6}^{-}$ in relation to the determined values of deposition rate for single-bead overlay welds made using the HARDFACE HC-O filler metal wire.

Experiment No. (n)	$\mathrm{Statistics}\;B_{6}^{+}$	$\mathrm{Statistics}\;B_{6}^{-}$
1	0.645	−0.355
2	0.667	−0.333
3	0.229	−0.771
4	0.700	−0.300
5	0.778	−0.222
6	0.859	−0.141
7	0.872	−0.128
8	0.500	−0.500
9	0.897	−0.103

**Table 7:** Analysis of the significance of the effect of the regression coefficients of the test object function.

Experiment No.	Variance of Measurement Results	Variance of Measurement Errors
n	s^2^(y)_i_	$s^{2}(y)=\frac{\sum_{i=1}^{9}s^{2}{\left(y\right)}_{i}}{9}$
1	0.0247	0.0423
2	0.0037	
3	0.0336	
4	0.0237	
5	0.0357	
6	0.1477	
7	0.0654	
8	0.0004	
9	0.0460	
**Absolute value of regression coefficient**	**Level of effect significance**	**Critical value of coefficient b_cr_**
|b_i_|	α	$b_{cr}=t_{cr}\sqrt{\frac{s^{2}(y)}{N\cdot r}}$
7.4804	0.01	0.1140
3.0189		
0.1967		
0.2078		
0.0344	0.40	0.0341
0.0600	0.15	0.0596

where: b_cr_—critical coefficient; t_cr_—critical value of Student’s *t*-test; N—number of tests (experiments); r—number of parallel measurements.

**Table 8:** Analysis of the adequacy of the test object function.

Experiment No.	Value of the Resultant Factor Calculated on the Basis of the Mathematical Model	Value of the Resultant Factor Obtained Experimentally	${\hat{y}}_{i}-y_{i}$	$({{\hat{y}}_{i}-y_{i})}^{2}$	Variance of Adequacy	Calculated Value of the F-Test
n	${\hat{y}}_{i}$	$y_{i}$			$s_{ad}^{2}(y)$	F
1	4.447	4.45	0.00	0.0000	0.1136	2.6830
2	7.318	7.42	−0.10	0.0100		
3	10.605	10.50	0.11	0.0121		
4	4.669	4.55	0.12	0.0144		
5	7.480	7.50	−0.02	0.0004		
6	10.707	10.81	−0.10	0.0100		
7	4.960	5.08	−0.12	0.0144		
8	7.711	7.59	0.12	0.0144		
9	10.878	10.88	0.00	0.0000		

## Data Availability

The research data was archived at the Department of Welding Engineering, Faculty of Mechanical Engineering, Silesian University of Technology.
